# Lysyl Oxidase Family Proteins: Prospective Therapeutic Targets in Cancer

**DOI:** 10.3390/ijms232012270

**Published:** 2022-10-14

**Authors:** Wei Wang, Xiangjun Wang, Feng Yao, Chao Huang

**Affiliations:** 1 Department of Hepatobiliary and Pancreatic Surgery, Shanghai Sixth People’s Hospital Affiliated to Shanghai Jiao Tong University School of Medicine, Shanghai 200233, China; 2 Thoracic Oncology Institute, Shanghai Chest Hospital, Shanghai Jiao Tong University, Shanghai 200030, China; 3 Department of Cell Biology, Medical School, Kunming University of Science and Technology, Kunming 650500, China

**Keywords:** lysyl oxidase (LOX), extracellular matrix (ECM), cancer, LOX family

## Abstract

The lysyl oxidase (LOX) family, consisting of LOX and LOX-like proteins 1–4 (LOXL1–4), is responsible for the covalent crosslinking of collagen and elastin, thus maintaining the stability of the extracellular matrix (ECM) and functioning in maintaining connective tissue function, embryonic development, and wound healing. Recent studies have found the aberrant expression or activity of the LOX family occurs in various types of cancer. It has been proved that the LOX family mainly performs tumor microenvironment (TME) remodeling function and is extensively involved in tumor invasion and metastasis, immunomodulation, proliferation, apoptosis, etc. With relevant translational research in progress, the LOX family is expected to be an effective target for tumor therapy. Here, we review the research progress of the LOX family in tumor progression and therapy to provide novel insights for future exploration of relevant tumor mechanism and new therapeutic targets.

## 1. Introduction

The lysyl oxidase (LOX) family is a secreted copper-dependent amine oxidase containing five family members: LOX; LOXL1; LOXL2; LOXL3; and LOXL4 [1]. Structurally, they have a highly conserved carboxyl terminus and a non-conserved amino terminus. In addition to the cytokine receptor-like (CRL) structural domain, the -C terminus contains a copper-binding motif and a lysyl-tyrosyl-quinone (LTQ) cofactor which affect the protein conformation and catalytic activity, respectively [2]. The -N terminus is highly differentiated except for the signal peptide region, which determines the differential function and tissue distribution. LOX and LOXL1 are secreted as inactive precursor proteins of pro-lysyl oxidase (pro-LOX) and pro-LOX-like protein 1 (pro-LOXL1), respectively, which can be further hydrolyzed by bone morphogenetic protein 1 (BMP-1) and related proteins into catalytically active proteins and the N-terminal pro-peptide region [2]. In the case of LOX, LOX mRNA is synthesized in the nucleus and translated into pro-LOX by translocation to the ribosome, then glycosylated in the Golgi apparatus, after which it is secreted to the surface of the cell membrane via transit vesicles and further sheared by peptidases into active LOX and LOX pro-peptide (LOX-PP) [3]. In contrast, LOXL2, LOXL3, and LOXL4 contain four scavenger receptor cysteine-rich (SRCR) domains which may participate in cell adhesion and protein interactions [4], and can mature without the hydrolysis process [5]. Therefore, the LOX family can be classified into two subfamilies, one consisting of LOX, LOXL1, and the other consisting of LOXL2, LOXL3, and LOXL4.

The LOX family mainly performs the biological function of mediating the covalent crosslinking of collagen and elastin in the extracellular matrix (ECM) by catalyzing the oxidative deamination of lysine residues to form peptidyl aldehydes [6], thereby enhancing the tensile strength and structural integrity of the tissue [7]. Moreover, its catalytic by-products, hydrogen peroxide (H_2_O_2_) and ammonia, also play essential roles in signal transduction. Thus, it plays an important role in maintaining growth and development of organisms (e.g., embryonic development, connective tissue function, and wound healing) [8]. Typically, the LOX family is secreted to the outside of the cell to regulate the ECM, and the extracellular enzymes can also be absorbed and returned to the cytoplasm/nucleus to regulate the cellular phenotype [9]. It is reported that LOX family members are widely distributed in human tissues where they have different expression patterns (Table 1), and the expression level of these proteins may vary at different stages of the development [10]. Experiments in knockout mice have demonstrated the differences in growth and development of organisms between the different family members. For example, LOX plays a significant role in the function and development of the cardiovascular system [11]; elastic fibers’ homeostasis in the skin, lung, large arteries, and other organs depends on LOXL1 [12]; loss of LOXL3 causes cleft palate and spinal deformities [13]. Many changes in the extracellular environment, such as hypoxia and hyperglycemia, can induce the dysregulation of its expression and lead to various diseases associated with ECM changes [10]. For instance, elevated activity or increased expression of LOX can result in atherosclerosis and scleroderma, and the decreased expression of LOX and LOXL1/3 can lead to pelvic organ prolapse and keratosis [9]. In addition, recent studies have found the aberrant expression or activity of the LOX family occurs in various types of cancer. It is widely involved in tumor invasion and metastasis, immunomodulation, proliferation, and apoptosis, all of which are promising targets for tumor therapy.

The table shows the top five human tissues with corresponding gene expression, listed in order of expression level (data from NCBI database).

## 2. LOX Family and Tumor Progression

At present, it is believed that the LOX family is involved in tumorigenesis and progression mainly through intracellular and extracellular pathways: Intracellularly, some members of the LOX family can not only modify specific proteins to regulate the transcription of tumor-associated genes, but also rely on their non-catalytical active structural domains or catalytic by-products H_2_O_2_ to activate relevant signaling pathways [9,14]; Extracellularly, tumor-associated ECM is often accompanied by increased collagen deposition. The overexpression of the LOX family will promote excessive collagen crosslinking and increase ECM stiffness, thus promoting tumor cell growth, invasion, metastasis, angiogenesis, resistance to cell death, and resistance to drug diffusion [9,15,16]. Ye et al. demonstrated the expression level and mutation type of the LOX family in different cancers using TCGA and cBioportal databases, and found that LOX, LOXL2, and LOXL4 genes frequently display mutation and overexpression in various cancers [8]. Overexpression of the LOX family is associated with hypoxia that is common in solid tumors, as the hypoxia-responsive element (HRE) exists in the promoter region of the LOX family, such as LOX, LOXL2, and LOXL4 [17,18]. However, it is unclear if the mutational status of LOX family members has an effect on their activity. Currently, research on the LOX family mainly focuses on LOX, LOXL2, and LOXL4, while LOXL1 and LOXL3 have been relatively less studied.

### 2.1. LOX

Initially, LOX was found to have the ability to inhibit the transformation of the H-Ras in mouse embryonic fibroblasts; therefore, it was considered as a tumor suppressor gene and named as Ras resection gene (Rrg) [19,20]. Subsequently, studies revealed that LOX-PP, but not LOX, inhibited the Ras-dependent cell transformation [21]. LOX-PP can decelerate Her-2/neu-driven tumor growth in the cancer xenograft model of nude mice by inhibiting AKT, ERK1/2, NF-κB activation, and epithelial–mesenchymal transition (EMT) [22]. In addition, LOX-PP can inhibit the transformation of H1299 lung cancer cells by decreasing B-cell lymphoma-2 (BCL-2) transcription [23]. Recently, more and more experiments have demonstrated that LOX mRNA and protein expression are significantly upregulated in various types of cancer, such as breast cancer, lung cancer, gastric cancer, and liver cancer; the high expression can promote tumor invasion and metastasis.

In breast cancer, compared with normal tissues, the overexpression of LOX in breast cancer tissues was associated with tumor volume, lymph node metastasis, and poor prognosis [24]; it also can be used as an evaluation indicator of poor prognosis in patients with estrogen receptor (ER)-negative breast cancer [25]. Moreover, studies have shown that LOX participates in the migration and tissue colonization of circulating breast cancer cells. Intraperitoneal injection of LOX inhibitors in mice significantly reduces the metastatic foci of breast cancer in both soft tissue and skeletal sites in the early stage of metastasis. However, it fails to improve tumor-induced osteolysis caused by established metastases [26]. For the mechanism by which LOX promotes tumor progression, Erler et al. found that the HRE in the LOX promoter is a downstream target of hypoxia-inducible factor 1 (HIF-1) [17]; meanwhile, LOX can also upregulate HIF-1α protein expression by activating the PI3K/Akt pathway. LOX and HIF-1 synergistically promote tumor proliferation and metastasis [27]. In addition, the Notch pathway and liver kinase B1 (LKB1) are also involved in HIF-1-dependent LOX regulation: the former increases the LOX gene transcription by promoting the recruitment of HIF-1 at the HRE of LOX, while the latter downregulates LOX expression by blocking the mTOR/HIF-1 axis [28,29]. In addition to interacting with HIF-1, intracellular LOX can activate the transforming growth factor (TGF-β1)-mediated p38 mitogen-activated protein kinase (p38 MAPK) pathway and the focal adhesion kinase (FAK)/steroid receptor-coactivator (Src) pathway in a H_2_O_2_-dependent manner to promote EMT in breast cancer cells [30,31]. Similarly, extracellular LOX can transduce the signal to downstream FAK/Src through integrin (ITG) receptors on the cell membrane surface and produce the same effect [32]. Furthermore, in breast and thyroid cancer cells, LOX interacts with the promoter of EMT transcription factor SNAIL2 to promote the transcription of SNAIL2, thereby increasing the secretion of tissue inhibitor of metalloproteinase 4 (TIMP-4) and enhancing the invasive and metastatic ability of tumors [33]. However, LOX silencing in breast cancer cells by siRNA can downregulate the expression of matrix metalloproteinase 2 (MMP-2) and MMP-9 in breast cancer tissues [24]. In metastatic breast cancer, the active LOX, typically found mainly outside the tumor cells, shows aberrant intracellular localization and enhances Akt, Erk, and NF-κB activities, promoting tumor proliferation and migration [22].

In lung cancer, the high expression of LOX associated with tumor infiltration is a prognostic marker for patients with early lung adenocarcinoma [34]. Liu et al. found that the relative mRNA or protein expression of LOX was notably higher in non-small cell lung cancer (NSCLC) tissues compared with adjacent/normal tissues, and the LOX expression was associated with MMP2, MMP9, tumor size, lymph node metastasis, pathological stage, and overall survival (OS) [35]. Moreover, LOX and HIF-1α share a similar expression level in NSCLC tissues, and the knockdown of LOX represses the invasion and migration ability of NSCLC cells induced by hypoxia [36,37]. As for the mechanism of its tumor-promoting effects, it was found that the reduction in serine/threonine protein kinase 4 (RIP4) expression can enhance STAT3 signaling in lung adenocarcinoma cells, which upregulates the LOX expression in an IL6-dependent manner and promotes dedifferentiation and metastasis of tumor cells [38]. Furthermore, the activation of PI3K/AKT, MEK/ERK, and SAPK/JNK downstream pathways of epidermal growth factor receptor (EGFR) can upregulate LOX expression, while the traditional anti-fibrosis drug silibinin represses EMT and ECM remodeling in NSCLC by blocking the EGFR/LOX pathway, thus inhibiting NSCLC invasion and migration [39]. Given the copper dependence of the LOX family, copper-transporting ATPase 1 (ATP7A) plays a pivotal role in the enzymatic activity of the LOX family. ATP7A silencing attenuates LOX activity and represses the growth and metastasis of mouse lung and breast cancer cells [40].

Currently, studies on gastric cancer (GC) have confirmed that the high expression of LOX is notably correlated with tumor infiltration depth, tumor differentiation, lymph node metastasis, vascular infiltration, and peritoneal metastasis, and patients with high LOX expression tend to have a poor prognosis [41]. Similarly, Han et al. found increased expression of LOX and HIF-1α in GC tissues, which can be regarded as a prognostic biomarker for patients with GC [42]. Peng et al., using a semiconductor quantum dot probe, found a higher expression of LOX in GC tissues than in para-cancerous tissues, and stromal activation (high density of macrophages, high density of microvessel, low neovessel maturation, intense expression of MMP-9, and low density of type IV collagen) induced by LOX overexpression in GC leads to a poor prognosis [43]. Studies have revealed that LOX in ECM may be a pivotal bridge between tumor-associated fibroblasts (CAFs) and tumor cells. TGF-β (most effective), platelet-derived growth factor (PDGF), stromal cell-derived factor-1 (SDF-1), and IL-1β induce fibroblasts to acquire the phenotype of CAFs and highly express the LIM structural domain protein Hic-5. Concurrently, the Hic-5 accumulated in the nucleus of fibroblasts induced LOX expression, which increased ECM stiffness and accelerated tumor progression [44,45]. In addition, LOX secreted by CAFs can promote tumor proliferation by enhancing the Warburg effect mediated by the AKT/p70S6K/HIF1-α pathway in tumor cells [46]. Recent studies have found that LOX participates in the construction of the tumor immunosuppressive microenvironment. HIF-1α-antisense strand 2 (HIF1A-AS2)/lncRNA (RP11-366L20.2)-miRNA-29c axis-mediated LOX overexpression may promote M2 macrophage polarization and immune evasion, thus enhancing the resistance of paclitaxel and fluorouracil in GC [47]. Furthermore, yes-associated protein 1 (YAP1) promotes LOX expression and secretion in glioma cells and drives macrophage infiltration into TME through activating the LOX/ITG- β1/proline-rich tyrosine kinase 2 (PYK2) pathway in macrophages; meanwhile, phosphoprotein 1 (SPP1) secreted by infiltrating macrophages can promote tumor growth and angiogenesis [48].

### 2.2. LOXL1

At present, there are relatively less studies on LOXL1 in tumors, and its effect on tumorigenesis and development remains controversial. Initially, LOXL1 and LOXL4 silencing by methylation were shown to have tumor-suppressive effects in human bladder cancer, while compensation experiments inhibited RAS-mediated ERK activation and reduced tumor-cell colony formation [49]. LOXL1 in colorectal cancer cells can repress tumor growth, invasion, and metastasis by increasing phosphorylation of the kinase MST1/2 to attenuate the transcriptional activity of YAP [50]. Similarly, Wang et al. found that LOXL1 knockdown can lead to the enhanced invasion and metastasis of prostate cancer cells by using the Genome-wide CRISPR-Cas9 screening [51].

However, the tumor-promoting effects of LOXL1 have also been reported successively in various types of cancer. LOXL1 overexpression enhances the invasiveness of lung cancer cells, and tumor cells with high LOXL1 expression yielding more cancer nodules through injection into mouse tail veins [52]. According to immunohistochemical analysis, the expression of LOXL1, monocarboxylate transporter (MCT) 1/2, and MMP-2/9 is higher in metastatic sites than that of the primary sites [52]. Similarly, papillary thyroid carcinoma (PTC) cells with high LOXL1 expression exhibit more aggressive behavior, in which LOXL1 can be used as a prognostic biomarker for advanced PTC [53]. Currently, the tumor-promoting mechanism of LOXL1 can be divided into two aspects of extracellular or intracellular effect: (1) Extracellular effect: TGF-β secreted by NSCLC cells promotes ITG α11 expression in CAFs via the Smad signaling pathway, which upregulates LOXL1 expression in CAFs, thereby inducing ECM remodeling and promoting the proliferation and invasion of NSCLC cells [54]. Furthermore, LOXL1 secreted by tumor cells can interact with other types of cells in TME and accelerate tumor progression. LOXL1 highly expressed in intrahepatic cholangiocarcinoma (ICC) can interact with FBLN5, an extracellular matrix glycoprotein containing the Arg-Gly-Asp (RGD) structural domain. Binding to ITG-αvβ3 on the surface of vascular endothelial cells (VECs) via the RGD structural domain, LOXL1, FBLN5, and the ITG-αvβ3 may form a complex, which regulates FAK and MAPK pathways in VECs and stimulates tumor angiogenesis [55]; (2) Intracellular effect: The vascular endothelial growth factor (VEGFR)/Src/CCAAT enhancer binding protein α (CEBPA) axis upregulates LOXL1 expression in glioma cells. LOXL1 can stabilize BAG family molecular chaperone regulator 2(BAG2) by blocking K186 ubiquitination and inhibit tumor apoptosis [56].

### 2.3. LOXL2

In recent years, more and more studies have confirmed its upregulated expression in breast cancer, liver cancer, gastric cancer, lung cancer, etc., in which LOXL2 plays a pivotal role in TME by promoting tumor-associated fibrosis and tumor angiogenesis [16]. Numerous clinical studies have also confirmed that high LOXL2 expression is related to tumor grading, poor prognosis, and reduced survival rate [13]. Although a few studies have shown the tumor suppressive effects of LOXL2, most of them cannot be verified in clinical samples [57].

In breast cancer, LOXL2 expression is negatively correlated with OS and metastasis-free survival (MFS) in patients with ER negative invasive breast cancer, whereas suppression of LOXL2 in mouse breast-cancer models impairs invasion and metastasis of breast cancer cells [58]. In tumor cells, LOXL2 can upregulate human epidermal growth factor receptor 2 (ErbB2) expression through the production of reactive oxygen species (ROS), and ErbB2-positive breast cancer patients with high LOXL2 expression have poorer OS and MFS [59]. In invasive ductal carcinoma (IDC) tissues, patients with high LOXL2 and oncostatin M (OSM) co-expression are more prone to metastasis [60]. OSM can contribute to ECM remodeling by upregulating LOXL2 expression in tumor cells, thereby enhancing the invasive ability of IDC cells [60]. Similar to LOX, the tight linkage between LOXL2 and CAFs is crucial for tumor progression: LOXL2 secreted by breast cancer cells can activate the FAK/Akt pathway in fibroblasts via ITG-β3 and promote fibroblast activation; subsequently, LOXL2 expressed by activated fibroblasts can further enhance tumor invasion and metastasis [61]. In addition, LOXL2 can also stimulate CAFs to secrete a high level of pro-lymphatic vascular factor (VEGF-C) and SDF-1α in a HIF-1α-dependent manner or directly activate the Akt-SNAIL and Erk pathways in lymphatic vessel endothelial cells, enhancing lymphatic vessel endothelial cell (LEC) proliferation and tumor lymphangiogenesis and promoting lymph node metastasis [62]. However, it has been suggested that LOXL2 may have a more prominent tumor-promoting effect in tumor cells: Hee et al. found that intracellular LOXL2 confers a greater invasive ability to tumor cells through the upregulation of SNAIL1-induced EMT compared with extracellular LOXL2-remodeling ECM [63]. In basal-like breast cancer cells, LOXL2 shows specific cytoplasmic/perinuclear expression and promotes tumor cell invasion and migration by repressing the transcription of cell polarity protein (Lgl2) and tight junction protein (claudin1) [64]. Similarly, in triple-negative breast cancer (TNBC) cells, LOXL2 silencing downregulates the expression of oxidation of H3 at lysine 4 (H3K4ox) and causes chromatin decompaction, leading to the sustained activation of the DNA damage response (DDR) and increased sensitivity of the chemotherapeutic drug, doxorubicin [65]. In addition, LOXL2 expression in dormant tumor cells (DTC) induces EMT and contributes to the acquisition of a cancer stem cell (CSC)-like phenotype, thereby causing the recurrence of breast cancer [66].

The high expression of LOXL2 can also be detected in tumor tissue and serum of hepatocellular carcinoma (HCC) patients, which is related to the poor prognosis of HCC patients [67,68]. Moreover, LOXL2 expression was positively related to tumor grading, metastasis, and vasculogenic mimicry (VM) [69]. LOXL2 knockdown using siRNA can decelerate the proliferation and colony formation of HCC cells, and promote cell cycle arrest and apoptosis [70]. In hypoxic environments, HIF-1α in HCC cells can directly affect LOXL2 and also upregulate LOXL2 expression through the TGF-β/Smad4 pathway [67]. Meanwhile, LOXL2 overexpression can promote intrahepatic metastasis and VM formation by interacting with SNAIL or increasing matrix stiffness [68,71]. Furthermore, LOXL2 in the extracellular matrix can also induce the recruitment of bone marrow-derived cells (BMDC) to the metastatic domain to promote extrahepatic metastasis [68]. In this regard, Wu et al. found that increased stromal stiffness can form a positive feedback loop with LOXL2 and accelerate pre-metastatic niche formation [72]. Increased stromal stiffness can upregulate LOXL2 expression and secretion in HCC cells by activating the ITG-β1/α5/JNK/c-JUN pathway, which subsequently promotes fibronectin (FN) production, MMP-9 and CXCL12 expression, and BMDCs’ recruitment [72]. In parallel, it can also induce the upregulation of HIF-1α, promoting LOXL2 expression and the polarization of M2 macrophages through activating the ITG β5-FAK-MEK1/2-ERK1/2 pathway [73]. In addition, it has also been reported that deubiquitinase (DUB) zinc finger RANBP2-type containing 1 (ZRANB1) can upregulate the transcription of LOXL2 and promote the proliferation and migration of HCC cells by deubiquitination of specificity protein 1 (SP1) [74].

It was found that the upregulation of LOXL2 expression is also associated with poor prognosis of NSCLC. The cytoplasmic and nuclear LOXL2 level is higher in lung adenocarcinoma (AD) and squamous cell carcinoma (SCC) than in adjacent normal tissues, and correlated with the p-TNM stage [75]. Cytoplasmic rather than nuclear LOXL2 level may be regarded as an independent prognostic factor in these patients [75]. Studies on the extracellular effect of LOXL2 have revealed that the MiR200/Zinc finger E-box-binding homeobox 1(ZEB1) induces LOXL2-mediated collagen stabilization and deposition in TME, which can activate the ITG-β1/FAK/Src pathway in lung cancer cells and promote tumor invasion and metastasis [76]. Increased collagen in lung cancer tissues can also bind to leukocyte-associated immunoglobulin-like receptor 1 (LAIR-1), which induces protein tyrosine phosphatase SHP-1-mediated T cell exhaustion, whereas inhibition of LOXL2 reduces tumor collagen deposition, increases CD8^+^ T cell infiltration, and the sensitivity of anti-PD-1/PD-L1 therapy [77]. Furthermore, NSCLC can develop immunosuppression in the hypoxic environment through the HIF-1α/LOXL2/EMT/tumor-infiltrating lymphocytes (TILs) axis, while HIF-1α inhibition combined with anti-PD-1 therapy can repress hypoxia-induced EMT, increase CD8^+^ T cell infiltration, and promote antitumor immunity [78].

Recently, there has been a new understanding of the pro-metastatic mechanism of LOXL2: circulating tumor cell (CTC) clusters can strengthen the metastatic ability of tumor cells [79,80]. The glycocalyx on the surface of CTCs can raise the survival rate of tumor cells during metastasis by enhancing the function of the cell surface ITG receptors [81]. In lung cancer cells, the downregulation of LOXL2 thickens the cell surface glycocalyx, which facilitates the formation of cell clusters and thus enhances the invasion and migration of tumor cells [82]. In fact, this is not contradictory to the previous tumor-promoting effect of LOXL2, because tumor cells in different regions are in diverse environments and have distinct biological behaviors. In the late metastatic stage, circulating tumor cell clusters are located in an environment more abundant in oxygen than before. Without the induction of hypoxia, the expression of LOXL2 is correspondingly reduced, which also facilitates the formation of cell clusters and enhances the migration of tumor cells.

### 2.4. LOXL3

In recent years, people gradually discovered the essential role of LOXL3 in tumor progression. For instance, a large cohort study of 597 primary GC cases showed that high LOXL3 expression was associated with GC infiltration and lymph node metastasis [83]. Correspondingly, the overall survival of LOXL3-positive patients is significantly poorer than those who are LOXL3 negative [83]. Similarly, LOXL3 expression is upregulated in HCC tissues and positively correlated with the infiltration level of multiple immune cells and the mRNA level of immune checkpoint genes; thus, HCC patients with high LOXL3 expression tend to have a poor prognosis [84]. Currently, the tumor-promoting mechanism of LOXL3 mainly can be divided into two points: (1) promote tumor invasion and metastasis: the interaction between LOXL3 and SNAIL in the perinuclear region prevents the SNAIL degradation and nuclear export, which inhibits the transcription of E-cadherin gene CDH1 and induces EMT [85]. Furthermore, the LIM-domain-only gene (LMO1) in neuroblastomas can promote extracellular matrix remodeling, focal adhesion complex assembly, and actin cytoskeleton rearrangement by upregulating the expression of ECM regulatory genes (LOXL3 and ITG), thus enhancing the invasion and metastasis of tumor cells [86]. Similarly, in invasive ductal breast cancer of no special type (IDC-NST), LOXL3 induces cross-linked bundling of collagen (increased thickness and network pore size) and promotes the collective invasion of frontier tumor cells [87]. This is actually consistent with the above-mentioned study that the downregulation of LOXL2 facilitates the formation of circulating tumor cell (CTC) clusters and enhances the metastatic ability of tumor cells, which indicates that LOX family expression in tumor tissues should have corresponding temporal and spatial differences; (2) Promote cell proliferation and inhibit apoptosis: LOXL3 is significantly upregulated in human melanoma, and patients with high LOXL3 expression frequently have a poor prognosis [88,89]. However, knockdown of LOXL3 causes melanoma cells to display an aberrant DNA damage response (DDR), leading to the accumulation of double-strand breaks (DSB) and aberrant mitosis, and ultimately inducing apoptosis due to increased genomic instability [88]. In addition, LOXL3 also has deacetylase/deacetyliminase activity, and LOXL3-mediated deacetylation in the nucleus disrupts STAT3 dimerization and eliminates its transcriptional activity. Knockdown of LOXL3 can upregulate the expression of proliferation-related genes, such as cell cycle protein D1 (CCND1), in a STAT3-dependent manner, thus the overexpression of LOXL3 in HCC cells can significantly delay the cell cycle and inhibit the growth of tumor cells [90,91].

### 2.5. LOXL4

Studies have shown that LOXL4 is extensively involved in tumor proliferation, metastasis, angiogenesis, and immunity. Vincent et al. found that LOXL4 was upregulated in metastatic peritumoral neutrophils of colon cancer liver metastases (CRCLM) with replacement histopathological growth pattern (HGP) compared with CRCLM with desmoplastic HGP and adjacent normal liver tissue [92]. It was suggested that neutrophils expressing LOXL4 might support the conversion of desmoplastic HGP to replacement HGP and be associated with a poorer prognosis of CRCLM with replacement HGP [92]. In addition, LOXL4 expression in circulating neutrophils from CRCLM patients is higher than in neutrophils isolated from healthy patients, and LOXL4 expression in neutrophils might serve as a novel biomarker in liquid biopsies [92]. In human HCC tissue, the upregulation of LOXL4 expression is correlated with vascular invasion, tumor differentiation, TNM stage, a poor prognosis [93], and high PD-L1 expression level [90]. Accordingly, most HCC patients with high LOXL4 expression and low CD8 expression have poor prognosis [94]. It was also found that, although LOXL4 overexpression promotes HCC cell invasion and migration, it has no effect on the proliferation of HCC cells [93]. At present, studies on the tumor-promoting mechanism of LOXL2 mainly focus on its extracellular effect: LOXL4 can be transferred between cells by HCC-derived exosomes, which can not only activate the FAK/SRC pathway in a H_2_O_2_-dependent manner to promote the migration of HCC cells, but also can be transferred to VECs to promote angiogenesis [94]. Furthermore, LOXL4 contributes to the formation of an immunosuppressive microenvironment. LOXL4 in HCC-derived exosomes can shape immunosuppressive macrophages through activating IFN-associated STATs-mediated PD-L1 [94]. In contrast, in HCC cells containing WT p53 (e.g., SK-Hep1, HepG2 cells), the DNA methyltransferase inhibitor 5-azacytidine relieves the transcriptional repression of LOXL4, and the accumulated LOXL4 can reactivate p53 and induce tumor cell apoptosis [95]. In this regard, Shao et al. suggested that LOXL4 silencing may free tumor cells from p53-dependent cell growth inhibition during the tumor initiation stage. Afterwards, p53 may lose its tumor suppressor function, which upregulates LOXL4 expression and promotes ECM remodeling and tumor metastasis [95].

It was also reported that the enhancer of zeste homolog 2(EZH2) in breast cancer cells represses the transcription of miR-29b and miR-30d by catalyzing H3K27me3, thereby promoting the expression of LOXL4 [96]. EZH2 is positively correlated with LOXL4 expression and tumor-associated macrophage infiltration (TAM), and LOXL4 knockdown can inhibit the proliferation and metastasis of breast cancer cells [96]. Moreover, overexpression of miR-328-5p, miR-210, and miR-135a-5p can promote the proliferation and migration of tumor cells by downregulating the expression of LOXL4 in NSCLC cells [97,98,99].

## 3. LOX Family and Tumor Therapy

At present, the tumor-promoting effects of LOX family (especially LOX and LOXL2) have been confirmed by more and more studies. Therefore, numerous LOX family inhibitors are being actively developed for targeted therapy, and their effects in immunotherapy, chemotherapy, and radiotherapy are gradually being explored.

### 3.1. Targeted Therapy

#### 3.1.1. β-Aminopropionitrile (BAPN)

BAPN is the first discovered pan-inhibitor of the LOX family with non-specificity and irreversibility [100]. BAPN can covalently bind to the LTQ cofactor in proteins to inhibit the catalytic activity of the LOX family [101], thus exhibiting the anti-cancer effect in therapeutic models of various types of cancer. For instance, Zhao et al. found that BAPN downregulated the expression of MMP2/9 in human gastric cancer cell BGC-823 by suppressing LOX activity, and inhibited tumor cell invasion and metastasis [102]. In the mouse model of peritoneal metastasis of ovarian cancer, BAPN reduces the number of peritoneal tumor nodules, total tumor weight, and collagen fiber amount [103]. In breast cancer, the inhibition of BAPN can significantly reduce the tumor volume [104] and diminish the metastatic colonization potential of circulating breast cancer cells [26]. Tzemah et al. suggested that the pharmacological inhibition with BAPN or LOX antibodies before breast cancer surgery can reduce the risk of lung metastases after surgery and increase the survival rate of mouse breast cancer models [105]. In addition, the inhibition of BAPN also impairs the invasion and migration of cervical cancer, hepatocellular carcinoma, pancreatic cancer, and undifferentiated thyroid cancer cells [16,106].

Although BAPN has a significant effect on tumor treatment, it is not conducive to preclinical optimization due to the lack of suitable chemical modification sites [106]. In previous clinical trials to treat hypertrophic fibrotic scarring and keloidal scars, BAPN was forced to terminate owing to its non-tumorigenic toxicity. Moreover, BAPN has been shown to have a teratogenic effect on developing zebrafish during the treatment process [107,108]. Therefore, the current use of BPAN is only for basic research and has not been recognized as suitable for clinical treatment.

#### 3.1.2. PXS Compounds

PXS compounds are the new generation of lysyl oxidase inhibitors. PXS-S1A, as the first generation of PXS inhibitor, is similar to BPAN in terms of activity and selectivity, but with plasticity. After further modification of PXS-S1A, the selective LOXL2 inhibitor, PXS-S2A, is developed [106]. PXS-S2A is reported to have good oral bioavailability, plasma stability, plasma protein binding, and high metabolic stability [109]. Chang et al. found that PXS-S1A and PXS-S2A can attenuate the proliferation, migration, and invasion of breast cancer cells in a dose-dependent manner [110]. In addition, other novel inhibitors of PXS have also been reported successively. PXS-S1C, a novel small-molecule inhibitor, can reduce LOXL2 expression in tumors and inhibit the growth and metastasis of oral cancer in the immunoreactive mouse model [111]. PXS-5153A, a novel dual LOXL2/LOXL3 inhibitor, can reduce the crosslinking and oxidation of collagen in a concentration-dependent manner. It has shown efficacy in liver fibrosis and myocardial infarction models and is expected to be applied in future tumor therapy [112]. PXS-5505 is in clinical trials for both myelofibrosis (NCT04676529) and hepatocellular carcinoma (NCT05109052) [113].

#### 3.1.3. PAT-1251

PAT-1251 (GB2064) is the first small-molecule irreversible inhibitor of LOXL2 to enter clinical trials with extremely low drug toxicity and low risk of forming reactive metabolites, and has great potential for development [114]. The phase I clinical trial of PAT-1251 in healthy subjects has confirmed that healthy subjects can tolerate this drug. A phase IIa study in subjects with myelofibrosis is now planned to discover its therapeutic value in anti-fibrosis [106]. Therefore, its anti-tumor effect deserves to be explored.

#### 3.1.4. CCT365623

Leung et al. developed a reversible inhibitor of LOX bearing an aminomethylenethiophene (AMT) scaffold, CCT365623, and proved that CCT365623 can significantly inhibit the growth and metastasis of LOX-dependent mammary tumors in the mouse model [115]. Moreover, CCT365623 can disrupt EGFR cell surface retention and delay the growth of tumor cells [116]. However, in view of its poor selectivity, the researchers replaced the previous thiophene core with a specific thiazole regioisomer, which made CCT365623 exhibit strong selectivity to LOXL2 and transform into an irreversible inhibitor [117]. This novel inhibitor may have a promising prospect in the future.

#### 3.1.5. AB0023 and Simtuzumab

The LOXL2-neutralizing antibody AB0023 can inhibit the enzymatic activity of LOXL2 by binding to the SRCR domain [101]. As the noncompetitive inhibitor, AB0023 can inhibit LOXL2 at any substrate concentration [101]. Although Finlay et al. demonstrated that AB0023 has incomplete inhibition of LOXL2 where BAPN has complete inhibition in vitro, AB0023 was superior to BPAN in reducing the metastatic burden of xenograft models [118,119]. The inhibition of LOXL2 by AB0023 can reduce activated fibroblasts, endothelial cells, and desmoplasia, inhibit the production of growth factors and the TGF- β pathway [119]. Moreover, the treatment with AB0023 can help normalize tumor blood vessels and improve the perfusion of tumor-associated vessels, thereby promoting the drug penetration into tumor tissues and increasing drug efficacy [120].

Simtuzumab is a humanized IgG4 monoclonal antibody of AB0023 [120]. Phase II clinical trials of simtuzumab have been conducted, such as the phase II clinical trial of simtuzumab combined with gemcitabine for patients with pancreatic cancer [121] and the Phase II clinical trial of simtuzumab combined with FOLFIRI (folic acid, fluorouracil, and irinotecan) as second-line therapy for patients with KRAS-mutant colorectal cancer [122]. In these clinical trials, simtuzumab was generally well tolerated, but its clinical benefit was limited, and some studies were terminated as a result [109]. It is thought that the collagen turnover in tumors is dynamic. Studies in colorectal cancer patients showed that collagen turnover fragments in serum increased with the tumor grade and that collagen turnover is higher in tumors compared to normal tissue [123]. However, Maller et al. suggested that the increased expression of collagen crosslinking enzymes and collagen crosslinks occurred early during malignancy. Although targeted drugs of LOX and LOXL2 can prevent further collagen crosslinking, they cannot reverse the crosslinks and collagen modifications already existing in ECM. This might explain why the therapeutic approaches targeting LOX and LOXL2 in advanced tumors cannot prolong the survival of patients [124].

#### 3.1.6. Copper Chelators

Tetrathiomolybdate (TM), a copper chelator, inhibits LOX family catalytic activity by inhibiting copper binding to the LOX family [7]. In the mouse model of breast cancer, TM inhibits lung metastasis but has no effect on primary tumors [125]. Accordingly, Chen et al. found that TM therapy, which is well tolerated and has a low incidence of toxicities, can reduce the recurrence rate of TNBC by inhibiting the endothelial progenitor cell (EPC)-mediated angiogenesis and LOXL2 in TME [125]. Similarly, TM also reduces the expression of nuclear factor-κB ligand (RANKL) in osteoblasts and osteocytes by inhibiting LOX activation, thereby reducing metastatic bone destruction due to head and neck squamous cell carcinoma (HNSCC) [126].

D-penicillamine (D-pen), commonly used in the past as a treatment for Wilson’s disease, non-selectively inhibits the catalytic activity of the LOX family at low concentrations [58]. In the mouse model of breast cancer, although D-Pen has no effect on tumor growth rate, it can reduce the lung and liver metastasis of tumors [58]. However, Siegel et al. suggested that D-pen can directly interact with collagen and prevent adjacent collagen crosslinking by forming thiazolidine rings, which is more effective than its indirect effect on ECM by inhibiting the LOX family [109].

Although TM and D-Pen have specific suppressive effects on tumors, they lack selectivity since copper ions function in various biological processes and involve different enzymatic reactions.

#### 3.1.7. Gallic Acid and Its Derivatives

Epigallocatechin gallate (EGCG), the main component of green tea polyphenols, is a trihydroxyphenolic compound that has been found to be a dual inhibitor of LOXL2 and TGF-β1 receptor kinase [127,128]. This compound induces LOXL2-specific lysine (K731) oxidation in a time-dependent manner, which irreversibly inhibits the catalytic activity of LOXL2. Therefore, despite poor bioavailability, EGCG is a potential therapeutic agent against tumorous or fibrotic diseases [127,128,129].

#### 3.1.8. Escin Ia

The saponin fraction of Aesculus chinensis Bunge fruits (SFAC) can attenuate the invasion and migration of TNBC cells [130]. The experiment showed that Escin Ia, the main effective component in SFAC, can reduce LOXL2 activity and promote E-cadherin expression, thereby inhibiting the EMT process of TNBC [130].

#### 3.1.9. Nanomedicine

With the advancements in targeted therapy research, people have gradually begun to focus on the complex tumor–matrix interactions [131]. Recently, Wei et al. found that stromal alignment is pivotal for drugs to penetrate the matrix barrier and exert their effects in tumor tissue. In this respect, Wei et al. designed enzyme-catalyzed responsive nanoparticles LOXL2-DDR1@MLP for pancreatic ductal adenocarcinoma (PDAC), which can release inhibitors of LOXL2 and discoidin domain receptor 1 (DDR1) in the tumor stromal to reduce the collagen crosslinking and remodel stroma topology. Consequently, the nanoparticles can improve the therapeutic effect by increasing drug penetration and accumulation in the PDAC tumor tissue [132].

### 3.2. Immunotherapy

Much more evidence indicates that the LOX family in tumor tissue is associated with immune cell infiltration in TME, which can shape the immunosuppressive microenvironment by activating EMT and lead to tumor immune escape. As mentioned above, the tight linkage between LOXL2, LOXL4, and PD-1 suggests that anti-PD-1 combined with targeting EMT therapy has a promising prospect. Peng et al. used the LOXL2 inhibitor ellagic acid (a dimeric derivative of gallic acid) combined with anti-PD-1 therapy to significantly inhibit the growth and metastasis of lung cancer and increase the CD8 T-cell infiltration in tumor tissue [78]. Similarly, BAPN combined with anti-PD-1 therapy also significantly delays the progression of pancreatic cancer, the combination therapy increases GrzmB CD8^+^ T cells and improves the ratio of CD8^+^ T to Treg cells, and cytokine detection in tumor supernatants shows an increased expression of Granzyme B, TNFα, and chemotactic cytokines RANTES [133]. This study suggested that LOX inhibition can improve ECM to reduce tumor stiffness, and subsequently facilitate T cell migration and improve anti-PD-1 efficacy [133].

As reported, the expression of the LOX family in immune cells is also of great significance. T cells stimulated by LOXL4-transfected dendritic cells (DCs) can secrete more IFN-γ, which may enhance the anti-tumor effect. This LOXL4-based tumor vaccine is expected to be adapted for patients with tumor-specific upregulation of LOXL4 [134]. In addition, paclitaxel chemotherapy can enhance rapid ECM remodeling and mechano-structural changes by inducing the expression of LOX in CD8^+^ T cells, subsequently improving the invasion and metastasis of breast cancer cells in the lung [135]. Hence, modulating LOX family expression in immune cells may be a novel strategy for tumor immunotherapy.

### 3.3. Chemotherapy

Ozge et al. used the chemotherapy-resistant (doxorubicin-resistant) mouse model of breast cancer to identify the molecular mechanism of TNBC chemoresistance. In the hypoxic TME, the HIF-1α-mediated transcriptional upregulation of LOX not only increases the expression of ITG-α5 and its ligand fibronectin in tumor cells, but also induces collagen crosslinking and fibronectin assembly, forming the mechanical barrier to prevent drug transport [136,137]. In addition, upregulation of LOX expression leads to activating the downstream FAK/Src pathway, inhibiting drug-induced apoptosis, and causing drug-resistance in TNBC [137]. Hence, chemotherapy combined with inhibitors targeting LOX (e.g., BAPN) or its downstream FAK (e.g., PF-562271)/Src (e.g., Saracatinib) can inhibit the development of TNBC chemoresistance and prolong patient survival.

### 3.4. Radiotherapy

Ionizing radiation can induce tumor cells to secrete LOX and enhance the invasive ability of naive tumor cells [138]. In addition, LOX can promote hypoxia-induced G2/M cycle arrest and repair of DNA DSBs in radiotherapy, indicating that LOX induces hypoxia-mediated radiotherapy resistance [139]. Radiotherapy also correlates with an upregulation of LOX and causes radiation-induced fibrosis, which is sensitive to LOX inhibitor (BAPN) [140]. Considering the essential function of LOX in radiotherapy, Cho et al. developed LOX-traceable nanoparticles (LOX_ab_-NPs) composed of LOX antibody (LOX_ab_) and paclitaxel. In the A549 lung cancer xenograft model, LOX_ab_-NPs^+^ exhibits particular tumor targeting in the radiotherapy group (above 7.0 times higher than in the non-radiotherapy group) and significantly delays tumor growth (LOX_ab_-NPs^+^ 222% vs. control >500%). This targeting vector based on the LOX antibody provides a novel insight for the future combination of radiotherapy and chemotherapy [141].

## 4. Conclusions and Perspectives

The LOX family participates in several fundamental processes in tumor progression, especially in invasion and metastasis, while proliferation, apoptosis, immunity, chemoresistance, and other aspects also have preliminary progress. However, more studies are still required because of the tissue-specific, spatial, and temporal differences in the expression of the LOX family. With the advancements in structural biology, the crystal structure of LOXL2 in a precursor state has been resolved [142]. Given the structural complexity of the LOX family, it can orchestrate different biological functions by interacting with proteins through multiple structural domains. Therefore, further analysis of the crystal structure of the LOX family and the development of novel inhibitors targeting its specific structural domains can precisely regulate aberrant biochemical reactions and facilitate the further dissection of the potential functions of the LOX family. In addition, considering the pivotal role of the LOX family in physiological processes, its systemic inhibition will inevitably cause numerous side effects. Therefore, developing novel delivery systems, such as enzyme-catalyzed responsive nanoparticles combined with specific enzyme inhibitors and standard cancer therapeutics, should be another direction for future exploration.

## Figures and Tables

**Table 1:** RNA-seq of lysyl oxidase in normal tissue samples.

LOX Isoform	Distribution
LOX	Fat, gallbladder, bladder, placenta, endometrium
LOXL1	Heart, prostate, placenta, fat, bladder
LOXL2	Placenta, fat, appendix, endometrium, bladder
LOXL3	Placenta, bone marrow, spleen, appendix, adrenal
LOXL4	Thyroid, testes, ovaries, endometrium, prostate
